# Conclusion: harmonisation in genomic and health data sharing for research: an impossible dream?

**DOI:** 10.1007/s00439-018-1924-x

**Published:** 2018-08-17

**Authors:** David Townend

**Affiliations:** Department of Health, Ethics and Society, and CAPHRI (Care and Public Health Research Institute), Maastricht University, Maastricht, The Netherlands

## Abstract

There are clear benefits from genomics and health data sharing in research and in therapy for individuals across societies. At the same time, citizens have different expectations and fears about that data sharing. International legislation in relation with research ethics and practice and, particularly, data protection create a particular environment that, as is seen in the articles in part two of this special issue, are crying out for harmonisation both at a procedural but at fundamental conceptual levels. The law of data sharing is pulling in different directions. This paper poses the question, ‘harmonisation, an impossible dream?’ and the answer is a qualified ‘no’. The paper reflects on what can be seen in the papers in part two of the special issue. It then identifies three major areas of conceptual uncertainty in the new EU General Data Protection Regulation (not because it has superiority over other jurisdictions, but because it is a recent revision of data protection law that leaves universal conceptual questions unclear). Thereafter, the potential for Artificial Intelligence to meet some of the shortcomings is discussed. The paper ends with a consideration of the conditions under which data sharing harmonisation might be achieved: an understanding of a human rights approach and citizen sensitivities in considering the ‘public interest’; social liberalism as a basis of solidarity; and the profession of ‘researcher’.

## Introduction

Knoppers and Joly started this special issue pointing to the great potential for genomic data and the understanding of genomic data’s part in revolutionising health care (Knoppers and Joly 2018). The Global Alliance for Genomics and Health, and research institutions around the world, have so many stories of how unlocking the power of data has changed understanding about disease, and paved the way to finding diagnosis, treatment, and even cures for conditions that until recently had only devastating prognoses. However, at the same time, Knoppers and Joly pointed to the need for common policies within which to realise the potential of this revolution not only on the individual story level, but as a story that is universal. In addition, the papers in this collection brought critical reflections on national responses to the question of international data linkage and harmonisation. The task I am asked to do in this third part of the collection is to reflect on whether harmonisation is an impossible dream. I am going to “stick my neck out” at the beginning of this reflection and say: harmonisation is possible, but it will not be found through a top–down harmonisation of national legislation, it will come from a bottom–up harmonisation of researcher practice and public acceptance.

In this paper, I will identify a number of key conceptual issues that are left open in the EU’s General Data Protection Regulation 2016/679 (GDPR) and suggest that these are common conceptual issues that must be resolved for effective data sharing both within and across national boundaries. These concern the development of legal safeguards to mitigate people’s concerns about the handling of their personal data in the research environment. I will also consider how far Artificial Intelligence can provide safeguards to mitigate risks to individual’s privacy. Thereafter, I will focus on the question of the public interest and the nature of privacy. I will consider what the public response ought to entail if we are to enjoy the benefits of the new understandings and technologies, and will end by briefly considering how the scientific community can help to develop the public discussion and public trust and confidence.

## What do we learn from the papers on national approaches?

The most striking thing to me in reading the papers in this collection is how, at once, the situation is so close to a coherent governance structure, and yet also so very far away. There is territorial diversity around a common legislative core. This is unsurprising, given the common origins of data protection (Phillips 2018) and the diverse contributions to governance from, particularly, pre-existing law on medical confidentiality, and privacy protection emerging in human rights law; the inclusion of the treatment of data within the Helsinki Declaration also introduces ‘ethics’ into the canon (World Medical Association 2013). From the international origins, jurisdictional (and in cases we have seen, territorial) differences have developed, and each jurisdiction sees a claim to a legitimate supremacy in the area, although none with such an extraordinary expression as the EU. Two things are interesting about the EU and the GDPR in this collection: first, that the need for compliance seems to be very much one-way (that ‘Third countries’ must comply to the EU’s approach), and second, the hint in Taylor, Wallace and Prictor’s paper (2018) that on Brexit the UK would no longer have a voice at the table—that the GDPR is an EU creation without external influence.^1^ If there is to be international harmonisation around sharing genomic data, then an international set of rules has to be found between jurisdictions.

The second observation is that whereas there are particular rules about data sharing between jurisdictions, and these are not harmonised, this is only part of the difficulty; there is conceptual and practical uncertainty and a lack of harmonisation within jurisdictions. Data sharing in genomic research is about linking data held in silos (institutions, research projects, biobanks, hospital records, general practitioner records, consumer companies, and other collections of lifestyle data). It seems from the experience of researchers and from the papers that are presented in this special issue and elsewhere that the rules are never straightforward for that sharing, be that between jurisdictions or within jurisdictions.

When considering data sharing between jurisdicitons, at the heart is the question of the provenance and conditions under which the data were gathered. In property law, there is a maxim “nemo dat quod non habet” (the “nemo data” rule): you cannot give what you have not got (Beale et al. 2012). This rings in the mind when reading the papers in this collection. Each jurisdiction, once the data are shared into the jurisdiction from outside, will have expectations about the provenance of the data. Will data gathered in a jurisdiction on the basis of ‘the public interest’ and without informed consent meet the requirements for processing in the new jurisdiction that requires informed consent as a condition for processing genomic data? Nemo data further bites, in the expectation of the data subject for a ‘reach-through’ of the conditions (particularly the consent conditions) under which the data were originally gathered. To go from one jurisdiction, where the data were gathered with consent, with perhaps expectations of re-consenting to refresh the consent in due time, to processing in a regime, where processing of the data is on the basis of the public interest (thereby breaking the link to the data subject’s consent) presents major difficulties. However, these are not simply problems created with transfers between jurisdictions, because it is equally clear reading the reports that the tensions are not settled within jurisdictions. Therefore, the route to data sharing harmonisation must include reform in national (and territorial) legislation, not simply in the cross-border transfer agreements.

This leads to a third observation: if there is to be any chance of harmonisation for genomic research (and arguably other biomedical or life science research using personal data), it will not be possible within *general* data protection laws. There is the real sense from the papers, and certainly from the GDPR, that treating the processing of personal data for research in the same way as processing for marketing or purely commercial practice causes major difficulties. There is a need to separate research from (other) commercial processing, to allow a different conceptual underpinning to emerge. This has two aspects: first, the conceptual details that require not just clarification, but philosophical agreement in data protection law, and second, the broader question of the public interest in genomic research.

## Specific conceptual uncertainties that are barriers to data sharing harmonisation

The GDPR is not an international standard; it is the latest iteration of data protection in the ‘supranational’ state of, currently, 28 Member States. It is influential, not least as it provides the terms of access to data in relation with those States (both within the EU and EEA and in its relation to non-EU States), and because it is a recent attempt at international negotiation of data protection and could be seen as a barometer of which issues are currently important in the negotiations. It is by no means perfect.^2^ The negotiations were protracted by any standard, and the range of disagreements was wide, particularly about balancing self-determination and autonomy-based privacy with broader public interest concerns about research. For our purposes here, however, its areas of unclarity and conceptual uncertainty are useful for articulating issues that an international consensus around data protection for genomic research should address.^3^

By way of introduction, the framework and content of the GDPR are very largely the same as Directive 95/46/EC (which is replaces). The bedrock of European data protection is a set of principles: to process the data fairly, lawfully, and in a transparent manner; that processing should be for a specified purpose; that the data collected should be only that required for the purpose and kept only for as long as is necessary for the purpose of the processing (and this includes the idea that one should seek to remove the identifiers from data as quickly as the purpose of the processing allows); that the data should be accurate; that the data should be kept securely; and that the data controller should ensure these principles in the processing of personal data.^4^

Routes to lawful processing are presented in two sets of conditions that must be met, one for processing data and one for lifting the ban on processing special personal data (including genomic data) (Articles 6 and 9, respectively). Alongside, the routes to lawful processing are the information provisions. Where data are gathered directly from the data subjects, they must be informed of the identify of the data controller (the person or institution that determines the purpose and nature of the processing of the personal data), and the purpose for which the data are processed (Articles 12 and 13). Where the data are not gathered directly, the provision is modified and the data subject must only be given the information, where it is not impossible or does not require a disproportionate effort (Article 14). The principles and routes to lawful processing create the duties on the data controller and are the safeguards for the data subject. The information provisions do not presume that informed consent is the route to lawful processing; rather, they provide the data subject with the necessary information to exercise their rights. Data subjects’ rights are contained in Articles 15 to 22: the right to access (to find out what the personal data relating to you that data controller holds) (Article 15); to rectification (Article 16); to erasure, the much-discussed ‘right to be forgotten’ [which, as Taylor et al. (2018) indicate, is restricted for processing for research purposes] (Article 17); to restrict processing (Article 18); to be notified about actions under Articles 16–18 (Article 19); to ‘data portability’ [which as Taylor et al. (2018) indicate extends only to those data the data subject has provided] (Article 20); the right to object to processing of one’s data (Article 21); and rights in relation with automated decision-making (Article 22).

Those familiar with Directive 95/46/EC will see the similarities between the two iterations of EU data protection. There is a strong argument that the GDPR is a stronger statement of established principles rather than a revolution. The regulatory environment is strengthened with the development of the European Data Protection Board (Article 68, et seq.), and the role of Data Protection Officers (Article 37); the GDPR increases the sanctions available for data breaches, and this is perhaps the single development in the law that has woken the community up to (EU) responsibilities that, arguably, it has had since 1995.

There is one area of important and original changes brought with the GDPR. The introduction of the “data protection impact assessment” (Article 35 et seq.) and the requirement to ensure “data protection by design and by default” (Article 25), when well-implemented, will change data protection considerably. Requiring data controllers to consider the impact of their proposed processing on the data subjects and to safeguard the interests appropriately is more than a cosmetic change, especially when coupled with the duties to declare data breaches (Article 33). These are significant developments that would sit well in any international data protection regime. However, there are elements that remain conceptually unclear. Unfortunately, for a Regulation (with direct effect in the Member States), there is a considerable amount of derogation and discretion left to the Member States (and particularly to the interpretations of the GDPR by their Supervisory Authorities), such that the goal of harmonisation across Europe still feels a considerable distance away. However, areas that remain unclear are a useful starting point for international discussions about the concepts that should operate for genomic data protection.

### Informed consent and anonymisation

The gold standard of medical ethics and the protection of participants in medical research has been informed consent and anonymisation. Genomic (and other medical research) causes difficulties for this approach. How far is it reasonable to require consent for every action performed in relation with personal data, especially in a (data sharing) research environment, where the gathering and connection of the data are for “research” or “medical research” but beyond that the purposes have very little specificity at the outset? Furthermore, there is increasing discussion about the perishability of consent (that consent is gathered for specific actions and for limited periods and should require refreshing over time to remain valid). Anonymisation (de-identification) is impossible in research that seeks to establish the longitudinal development of the individual’s life story—as is often the case in genomic research. It is equally impossible to operate in a de-identified way when one seeks to link data sets and connect information about particular individuals (or guard against double counting of individuals in the results). The context of medical confidentiality is also changing with the development of precision medicine; our expectations about medical treatment that is increasingly personalised will require greater linkages of data. One of the foreseeable expectations in such a changed situation will be the link between therapeutic and research data, with participants expecting to see the issues raised in their research participation linked to their general health data portfolio. Again, anonymisation does not operate in such an environment and the gold standard moves to pseudonymisation of data to ensure interoperability and portability for patient benefit.

Informed consent and anonymisation, however, remain extremely important values in the GDPR (and the data protection in other jurisdictions), because for most personal data processing applications other than (genomic and medical) research, self-determination and autonomy are the most important values in play. There is a question, even in the areas of marketing or social media, whether informed consent is a sufficient safeguard (because the data subject is relatively powerless to audit the processes of commercial institutions). The imperative towards de-identification of individuals within data sets, seen in Articles 5(1)(e) and 89, extends only as far as the purpose of the processing allows; anonymisation seems to be rather ruled out in genomic data sharing by the requirements of the purpose. This has to be acknowledged in the law; genomic research should not be seen as standing against the intention of the law in this respect.

As has been noted throughout this special issue, the GDPR has left the question of consent for genomic and medical research unclear. In the Articles 4(11), 6(1)(a), 7, and 8, informed consent is established as a route to lawful processing. The Articles indicate that a narrow or specific reading of informed consent is required.^5^ Recital 33 of the GDPR creates an opportunity, a realisation, that broad consent is necessary for the operation of medical research.^6^ This tension between the Articles and Recital must clearly be resolved centrally to facilitate data sharing. However, as will be discussed later in relation with the public interest, the distinction between narrow and broad consent is not one that can be successfully resolved by a legislative statement.

### Compatible processing

Where personal data are gathered for one stated purpose (perhaps using a rather narrow construction of informed consent), it is unclear how far those data can then be used for other ‘compatible’ purposes within the terms for which the data were originally gathered. Using a “processing for a compatible purpose” construction is, of course, very attractive for researchers (and in the GDPR it seems as if research is given special treatment to use this route):^7^ already gathered data can be processed for research purposes within the terms of the original gathering of the data, even when it was not one of the originally stated purposes for the processing. Therefore, if I gather data for, for example, diagnostic purposes, under the GDPR scientific research on those data would be a compatible purpose for processing the data, even if it was not a stated purpose when I gathered the data (but one must stress—under the GDPR). Whereas there is greater clarity in the GDPR than in Directive 95/46/EC on the availability of processing for compatible purposes, the extent to which data controllers (researchers) will be able to rely on this is not clear. Again, this is an area, where a clear stance is required in legislation regarding genomic (and other medical) research. First, this is because of the interplay of different legal requirements relating to the data. Where the data to be shared are medical data (gathered, for example, by a doctor), then it is likely to be subject to limitations on access and sharing under, for example, other confidentiality laws^8^; data protection permission will not be sufficient to overcome other supervening legal duties over the data.^9^ Second, the possibility for processing for compatible purposes might also be limited by the terms on which the data have been gathered in the particular case; the drafting of the consent may remove the appeal to the general data protection law principle. Third, the research ethics committee could require, following Article 32 of the Helsinki Declaration, consent for research. The derogation available in Article 32 is only the exceptional circumstance, where obtaining consent would be impossible or impracticable and the research is regulated by a research ethics committee; the GDPR safeguards could contribute to ensuring a suitable environment to accept the derogation, but it is, arguably, a narrow derogation. With these three difficulties, the availability of processing the data for research as a compatible purpose to the stated original purpose(s) requires an accepted legislative provision^10^ to which researchers can appeal in different circumstances beyond relying on making a strong argument on a case-by-case basis.

### Re-identification of data

This occurs in two sorts of problem. Imagine first a biobank which has gathered samples and medical histories from a number of volunteers for stated research purposes. The biobank holds these data in a pseudonymised form, because the purpose of the biobank is to develop longitudinal data about individuals. It makes the data available to (authorised) researchers, however, in a de-identified form, with no provision for providing the key; the researchers cannot identify particular individuals within the data set. Because the data remain identifiable in the hands of the biobank, it is not clear in the GDPR if the data are de-identified in the hands of the researcher. That is a first version of the problem. A second is imagining the same scenario outside a closed environment; the researcher has other data and access to other data sets that when combined with the de-identified data from the biobank could re-identify individuals within the data sets. In some jurisdictions, for example, the USA, the data in these scenarios are de-identified data; elsewhere this is not clear.

Data are, under Article 4(1), personal data under the GDPR when they relate to an “identified or identifiable natural person” “who can be identified directly or indirectly”. Recital 26 provides two useful indications of how that should be interpreted: “[t]o determine whether a natural person is identifiable, account should be taken of all the means reasonably likely to be used” and, “[t]o ascertain whether means are reasonably likely to be used to identify the natural person, account should be taken of all objective factors, such as the costs of and the amount of time required for identification, taking into consideration the available technology at the time of the processing and technological developments”. The question remains how Supervisory Authorities, Research Ethics Committees, and the European Data Protection Board will interpret this. There is a very good argument that the agreements for the transfer of data (Article 28) could be the key to this. The written agreement concerning the transfer of data from the data controller could include undertakings about not re-identifying individuals, or what to do in case of accidental re-identification; it could create a duty of confidentiality to the recipient of the data, with sanctions for breach. This, arguably, could meet the requirements in a ‘reasonably likely’ assessment. However, again, this requires harmonisation and acceptance in the governance community to work.

## Building a bottom–up response to harmonisation: the contribution of AI

Returning to the overall question, ‘is harmonisation an impossible dream?’, and having read the paper so far, one might conclude that it is an impossible dream. However, what has been presented so far is about a ‘top–down’ solution to harmonisation. The issues addressed do need clarification, but the genomic research community does not have to rely on international organisations and inter-governmental initiatives to find those solutions. There is a much greater opportunity here for ‘bottom–up’ harmonisation from the community itself, not least, because there is such a lack of clarity in the governance structures, but also because there is such a desire to realise the potentials of genomic (and other medical) research internationally.

The brief overview of the GDPR above indicated that, in the development of the Data Protection Officer role, the inclusion of impact assessments, and the concept of data protection by design and default, there is a lot of responsibility placed on the Data Controller. With the opportunity for sectoral codes of conduct under Article 40 (and the opportunities of ISOs generally), different communities, internationally, can find clarity and practice that meets the generally expressed imperative for safeguards, and the expressed hopes for advances in genomics and medical science. We have achieved, for example, through the work of GA4GH explained elsewhere in this collection, harmonisation of technical standards for building data sets. Artificial Intelligence (AI) can provide a next level of (international) harmonisation, this time in no small part for governance as well as for data science purposes.

The FAIR principles (Wilkinson et al. 2016)—that the architecture of data processing in data science should be designed to ensure that the data are Findable, Accessible, Interoperable, and Reusable—are becoming an internationally recognised standard, as much from the community of users as from more institutional backers. FAIR must operate in compliance with law and ethics, so it is not a solution to the questions posed in this special issue, but it does show the power of the community to solve its difficulties. There are further examples of how AI might help us.

The Personal Health Train^11^ seeks to create a federate data processing tool for data-sharing research (Damiani et al. 2018; Deist et al. 2017; van Soest et al. 2018; Roelofs et al. 2014). The personal health train project is developing a way of interrogating data at its location, without requiring the data to leave the institution. Only answers to the questions posed are given to the researcher. This reduces the risks (and concerns) about moving data sets to centralised locations. Data hubs have equally been developed that do move large data sets together into particular secure locations, creating security through highly regulated access and a closed environment—where, for example, the data science tools available for the processing of the data are only provided by the hub. The benefit of these developments is that they can be created with a high level of data security, and with a high level of data protection based on defensible solutions to the different legislative problems identified, without having to harmonise the international law and ethics approach.

“Blockchain”, and subsequent technologies in that ilk, could also be very important in achieving a harmonised data sharing environment. Blockchain offers a secure record of transactions. Its security comes from the storage of the record on multiple sites, making the possibility of changing the record (in the current computing reality) almost impossible (Voshmgir and Kalinov 2017). This technology, coupled with truly dynamic consent portals for participants, could have enormous significance for the governance of data sharing. Not only will individuals be able to make effective and granular choices about their interaction with data sets, more importantly, they will be able to see what has happened to their data. The revolution is plain: we can move from trust alone to trust with proof. Whereas the interaction between people and their personal data has depended upon only trust, there is the possibility in the new AI technologies that people will be able to rely on proof. Until now, signing up to participate in a research project, or the building of a data set (or, being included in a data set in the public interest) has depended completely on trusting the professionals to do as they promise, with little opportunity to ensure that the promises were kept (either for the individual participant or for, for example, the research ethics committee approving the research). By defining the ‘transactions’ that are recorded in the Blockchain, builders of data sets will be able to give people the ability to see with confidence who has had access to their data; that, for example, insurance companies have not had access to the data set.

## From the bottom–up: public Interest, professionalism, and discourse

Law and ethics clarifications and AI tools will undoubtedly provide new solutions to the governance difficulties that we face around data sharing. However, of themselves, they will not be sufficient. At different points in this special issue, authors have pointed to the ‘public interest’ as a route to lawful processing that can alleviate difficulties of, for example, informed consent. There are purposes for the processing of personal data that transcend the wishes of the individual and that can be carried out in the public interest. I have argued elsewhere that this can be constructed not only on the basis of Utilitarian balancing of the greatest utility for the greatest number, but also through Kant’s Categorical Imperative—that the individual in making claims about their privacy in relation with the use of their data must be mindful of the effect of that claim (which is not a claim to a fundamental but secondary right) on others, and that one cannot instrumentalise others by making a privacy claim as one must treat others as ends in themselves and not merely as means to one’s ends (Townend 2017).

Processing in the public interest will not be a sufficient solution to the data sharing problem. There are two aspects that are important to draw out in realising the dream of data sharing harmonisation: rationality and professionalism. Throughout this special issue, the tension between autonomy and solidarity can be felt, and this is especially the case in relation with the appeal to the public interest. Simply making the appeal—telling people that it is legitimate that their data are or were processed in the public interest (or that they must accept a broad rather than an narrow consent) will not, for many, be sufficient. When one looks at public opinion surveys, particularly, for example, the Eurobarometers on biotechnology and data protection, one can see that respondents indicate a range of sensitivities from those who are very happy that their data be used in research to those who are extremely unhappy that their data should be used in research.^12^ An element of this that is difficult for health research to reconcile is a range of sensitivities about the involvement of commercial interests in processing personal data (again, some are happy, others unhappy) as heath research often has a commercial context. An appeal to the public interest is not one that some of the public will easily accept and, unlike, for example, national security, where a robust approach is taken, a more sensitive approach is needed in relation with health matters, as the individual’s response to unease about participation may well be not seeking healthcare early. Genomic (and broader) health research must ensure that its data sharing solutions do not encourage a two-tier approach of those who participate in healthcare and those who do not.

Some might say that data protection laws, including the GDPR,^13^ do not help by blurring the distinction between ‘pure’ and ‘applied’ research and that the genomic and medical research that we are discussing in relation with this data sharing will end in the development of new therapies in the commercial sector. Knoppers et al. (2014) have addressed this, to some extent, in their arguments that the governance of data sharing must be based on all the principles in the human rights canon. They argue strongly that privacy is not the only right, but that also the right to share in the scientific benefits of one’s community and to be recognised as the author of scientific developments is equally important. There is a further argument that the human rights to health and to property also play important roles in framing the discussion about the involvement of commerce in data sharing (UN General Assembly 1948, Articles 17 and 27; UN General Assembly 1966, Article 12). The four rights taken together, and interpreted through the lens of, for example, Kant’s Categorical Imperative not to instrumentalise people, produces a balance that must allow for more than the current ‘altruism in, profit out’ paradigm, but at the same time must accept that without much greater social revolution, the translation of (medical) scientific advances will be through commercial pharmaceutical industries. There is, however, a space for those industries to recognise the need for greater transparency in their claim to, for example, the calculation of costs and the fairness of profits, and to accept principles of benefit sharing in access to medicines for those who individually or in communities participate in their research.

This discussion points to a need for a further discourse in society: what is an appropriate balance between autonomy and solidarity in data sharing? I do not propose an answer, but merely make an observation. When one is given a choice, is it a perfectly free choice without constraint? For example, in our context, is the question, will you allow your data to be shared, a perfectly free choice? The first aspect of this is that it feels like an answered question in liberalism. Since, perhaps, the 1980s and its iteration of economic liberalism, Hayek’s rejection of social liberalism seems to have great resonance with many; it is acceptable to say that I have a free choice about, for example, participation in a biobank or allowing my already gathered data to be used in research, regardless of whether or not I am likely to suffer a harm. My privacy right is, for many, an absolute right, based in a reading of (economic) liberalism that rejects any social duties upon me. It is worth noting that this is a change from expressions of liberalism that allow for social liberalism. Adam Smith’s economic theory is based on his moral theory that places duties between individuals; Locke’s theory of property depends on sufficient resource being left for others; Kant’s Categorical Imperative recognises the duty to others; and John Stuart Mill whilst recognising the central importance of the liberty of the individual acknowledges that it is limited by the duty not to harm others. In general, this would be an insignificant debate, but in data sharing for genomic and health research, it is crucial. Many people seem to operate with something of an inconsistency: I want almost absolute privacy, but at the same time when I need healthcare, I want the carers to be able to cure me. These cannot be reconciled easily, especially in the climate where social liberalism is rejected, but the creation of a data-sharing environment requires public debate on these hard issues.

## Future steps

The thrust of this paper has been that there are a number of areas that require clarification in the law and that these are better solved, especially given the opportunities offered by AI, through a bottom–up approach. I have also suggested that a shift to embracing the idea that this data sharing is more a matter of the public interest than, for example, informed consent, requires a high degree of public engagement. This is, in part, because the public’s hopes and fears for the use of their personal (health) data are not universal and there is a considerable range of concerns that have to be taken into account, but also because there is an underlying difficulty in appealing to simple solidarity to many who embrace more individualist autonomy.

The question is, therefore, where might these discussion be had? Perhaps, the answer lies in a continuation of the work that already emerging. Organisations such as the Global Alliance for Genomics and Health (GA4GH) has made great steps in bringing together the community of researchers. It would seem that the public might be helped in its acceptance of data sharing if the scientific community took its self-regulation to a more formal level: a professional accreditation of researchers in the field, formally adhering to a code of conduct, with continuing education to develop the profession of data sharing in research, and including applied researchers. Such a community could become a place, where two things could happen: first, different publics across the world could be engaged in a discussion about data sharing, with members of the profession explaining what data sharing work entails, what the risks are, and how those risks can be mitigated in safeguards; and second, the publics’ responses in the engagement can be developed into governance structures. Is genomic and health data sharing an impossible dream? No, if public and professional discourse is raised to a new level of seriousness.

## Footnotes

1 This, of course, could be part of the divorce argument, but the previous approach to Safe Harbor might indicate more of a trend in the approach.

2 Indeed, in a contribution to a previous special issue in the area, many of the issues raised in this paper were indicated as problematic but remain unresolved (Townend 2016).

3 GDPR Article 40 allows for sectoral codes of guidance to be developed and approved. BBMRI-ERIC is working towards such a code for medical research, with the aim of developing clarity around which consensus can be built (Litton 2017).

4 These are the GDPR Article 5 principles of ‘lawfulness, fairness and transparency’, ‘purpose limitation’, ‘data minimisation’, ‘accuracy’, ‘storage limitation’, ‘integrity and confidentiality’, and data controller ‘accountability’.

5 Article 4(11) “‘consent’ of the data subject means any freely given, specific, informed and unambiguous indication of the data subject’s wishes by which he or she, by a statement or by a clear affirmative action, signifies agreement to the processing of personal data relating to him or her”.

6 Recital 33 “It is often not possible to fully identify the purpose of personal data processing for scientific research purposes at the time of data collection. Therefore, data subjects should be allowed to give their consent to certain areas of scientific research when in keeping with recognised ethical standards for scientific research. Data subjects should have the opportunity to give their consent only to certain areas of research or parts of research projects to the extent allowed by the intended purpose.”

7 GDPR, Articles 5(1)(b) and 6(4) and in Recital 50.

8 For example, the Dutch Medical Contracts Law (the Wet op de geneeskundige behandelingsovereenkomst) Sect. 7.457 imposes a duty of medical confidentiality that would not include a transfer for research purposes (without specific consent).

9 See, for example, the reference to confidentiality in the HRA comment consent and the public interest under the GDPR: https://www.hra.nhs.uk/planning-and-improving-research/policies-standards-legislation/data-protection-and-information-governance/gdpr-guidance/what-law-says/consent-research/ (last visited: 16 July 2018).

10 Particularly one that brings the Helsinki Declaration more into line with international data protection law expectations in research.

11 http://www.personalhealthtrain.org (last visited: 16 July 2018).

12 http://ec.europa.eu/commfrontoffice/publicopinion/index.cfm (last visited: 16 July 2018) See, particularly, on data protection, Eurobarometers numbers 147 and 196 (2003), 225 and 226 (2008), 359 (2011), and 431 (2015), and on biotechnology, Eurobarometers numbers 61 (1991), 80 (1993), 108 (1997), 134 (2000), 177 (2003), 244b (2006), and 341 (2010).

13 See Recital 159.

## References

[CR1] Beale HG, Bridge M, Gullifer L, Lomnicka E (2012). The law of security and title-based financing.

[CR2] Damiani A, Masciocchi C, Boldrini L, Gatta R, Dinapoli N, Lenkowicz J, Chiloiro G, Gambacorta M, Tagliaferri L, Autorino R, Pagliara M, Blasi M, van Soest J, Dekker A, Valentini V (2018). Preliminary data analysis in healthcare multicentric data mining: a privacy-preserving distributed approach. J e-Learn Knowl Soc.

[CR3] Deist TM, Jochems A, van Soest J, Nalbantov G, Oberije C, Walsh S, Eble M, Bulens P, Coucke P, Dries W, Dekker A, Lambin P (2017). Infrastructure and distributed learning methodology for privacy-preserving multi-centric rapid learning health care: euroCAT. Clin Transl Radiat Oncol.

[CR4] Health Research Authority (2018) Consent in research. NHS. https://www.hra.nhs.uk/planning-and-improving-research/policies-standards-legislation/data-protection-and-information-governance/gdpr-guidance/what-law-says/consent-research/. Accessed 16 July 2018

[CR5] Knoppers BM, Joly Y (2018). Introduction: the why and whither of genomic data sharing. Hum Genet.

[CR6] Knoppers BM, Harris JR, Budin Ljsne I, Dove ES (2014). A human rights approach to an international code of conduct for genomic and clinical data sharing. Hum Genet.

[CR7] Litton J-E (2017). We must urgently clarify data-sharing rules. Nature.

[CR8] Phillips M (2018). International data-sharing norms: from the OECD to the general data protection regulation (GDPR). Hum Genet.

[CR9] Regulation (EU) 2016/679 of the European Parliament and of the Council of 27 April 2016 on the protection of natural persons with regard to the processing of personal data and on the free movement of such data, and repealing Directive 95/46/EC (General Data Protection Regulation). Official Journal of the European Union L 119/1. http://data.europa.eu/eli/reg/2016/679/oj. Accessed 16 July 2018

[CR10] Roelofs E, Dekker A, Meldolesi E, van Stiphout RGPM, Valentini V, Lambin P (2014). International data-sharing for radiotherapy research: An open-source based infrastructure for multicentric clinical data mining. Radiother Oncol.

[CR11] Taylor MJ, Wallace SE, Prictor M (2018). UK: transfers of genomic data to third countries. Hum Genet.

[CR12] Townend D (2016). EU laws on privacy in genomic databases and biobanking. J Law Med Ethics.

[CR13] Townend D, Capps P, Pattinson SD (2017). Privacy, politeness and the boundary between theory and practice in ethical rationalism. ethical rationalism and the law.

[CR14] UN General Assembly (1948) Universal declaration of human rights (217 [III] A)

[CR15] UN General Assembly, International Covenant on Economic, Social and Cultural Rights, 16 December 1966, United Nations, Treaty Series, vol. 993, p. 3

[CR16] van Soest J, Sun C, Mussmann O, Puts M, van den Berg B, Malic A, van Oppen C, Townend D, Dekker A, Dumontier M (2018). Using the personal health train for automated and privacy-preserving analytics on vertically partitioned data. Stud Health Technol Inform.

[CR17] Voshmgir S, Kalinov V (2017) Blockchain: A Beginners Guide. BlockchainHub. https://onedrive.live.com/?authkey=%21ANKo0dRYCNYSQKU&cid=1CB2899A0E42236C&id=1CB2899A0E42236C%21130359&parId=1CB2899A0E42236C%21130358&o=OneUpAccessed 20 July 2018

[CR18] Wilkinson MD, Dumontier M (2016). The FAIR guiding principles for scientific data management and stewardship. Sci Data.

[CR19] World Medical Association (2013). Declaration of Helsinki: ethical principles for medical research involving human subjects. JAMA.

